# Computational analysis of CD40L missense variants A123E, S222F, and G257R predicts altered CD40 binding and trimeric stability in X-linked hyper-IgM syndrome

**DOI:** 10.3389/fimmu.2026.1861005

**Published:** 2026-08-17

**Authors:** María Guadalupe Velásquez-Ortiz, Eduardo Jardón-Valadez, Raúl Fernando Reyes-Huerta, Jovani Catalán-Dibene, Laura Berrón-Ruiz, Gabriela López-Herrera, David Eduardo Meza-Sánchez, José Luis Maravillas-Montero

**Affiliations:** 1 Departamento de Medicina Molecular y Bioprocesos, Instituto de Biotecnología, Universidad Nacional Autónoma de México, Cuernavaca, Morelos, Mexico; 2 Posgrado en Ciencias Biológicas, Universidad Nacional Autónoma de México, Unidad de Posgrado, Ciudad Universitaria, Coyoacán, Mexico City, Mexico; 3 Departamento de Recursos de la Tierra, Universidad Autónoma Metropolitana, Estado de México, Mexico; 4 Doctorado en Ciencias Biomédicas, Universidad Nacional Autónoma de México, Unidad de Posgrado, Ciudad Universitaria, Coyoacán, Mexico City, Mexico; 5 Division of Biomedical Sciences, School of Medicine, University of California, Riverside, Riverside, CA, United States; 6 Laboratorio de Inmunodeficiencias, Instituto Nacional de Pediatría, Coyoacán, Mexico City, Mexico; 7 Red de Apoyo a la Investigación, Universidad Nacional Autónoma de México-Instituto Nacional de Ciencias Médicas y Nutrición Salvador Zubirán, Mexico City, Mexico

**Keywords:** binding free energy, CD40, CD40L, inborn errors of immunity, missense mutations, molecular dynamics, X-linked hyper-IgM syndrome

## Abstract

**Introduction:**

CD40L (CD154) is a homotrimeric member of the TNF superfamily protein expressed on activated CD4+ T cells whose interaction with CD40 is required for class-switch recombination, somatic hypermutation, and B cell terminal differentiation. Loss-of-function mutations in gene CD40LG cause X-linked hyper-IgM syndrome type 1 (HIGM1), characterized by recurrent infections, lack of IgG, IgA and IgE, and near-normal or elevated IgM.

**Methods:**

We implemented computational strategies to describe the structure and dynamics of the CD40L-CD40 complex for the native protein and three missense variants not previously characterized at the molecular level: A123E, S222F and G257R. Two independent 300 ns simulations were run per system in explicit solvent at 300 K and 0.15 M NaCl. In addition, we evaluated the energetics and electrostatics of the complex using continuum solvent models, such as molecular mechanics-Poisson-Boltzmann Surface Area (MM-PBSA) and Adaptive Poisson-Boltzmann Solver (APBS).

**Results:**

We found that A123E caused a moderate, energetically coherent reduction in binding affinity consistent with partial loss of function. S222F caused the largest reduction in binding free energy (weakest binder by MM-PBSA) along with an increase of the CD40L-CD40 contact area, consistent with a geometrically expanded but energetically disrupted interface where the aromatic side chain offsets paradoxically favorable electrostatic reorganization. G257R maintained near-native binding free energies but reduced the trimeric contact area by 20-22%, and increased the radius of gyration of CD40L, consistent with structural loosening of the homotrimer that we predict could uncouple binding competence from signaling competence, with potential effects on CD40 receptor mobility and mechanotransduction at the immunological synapse that remain to be tested experimentally.

**Discussion:**

These findings may offer a structural basis for the clinical heterogeneity of HIGM1 and illustrate what computational approaches can contribute to the interpretation of CD40LG variants, including novel variants identified in Latin American patients where experimental validation is rarely available.

## 1 Introduction

The CD40L-CD40 (CD154) axis is one of the most critical costimulatory checkpoints in adaptive immunity. CD40L belongs to the TNF superfamily and CD40 to the TNF receptor superfamily; their interaction activates canonical and non-canonical NF-κB pathways as well as MAPK, PI3K/AKT, and JAK3/STAT signaling (1). In B lymphocytes, this interaction drives germinal center formation, immunoglobulin class-switch recombination (CSR), affinity maturation, and the generation of long-lived plasma cells and memory B cells. In dendritic cells and macrophages, CD40 ligation upregulates MHC-II, co-stimulatory molecules, and pro-inflammatory cytokines including IL-12 and TNF-α, bridging humoral and cell-mediated responses. CD40 is additionally expressed on endothelial cells, smooth muscle cells, and certain tumor cells (2, 3); so the CD40L-CD40 interaction coordinates immune signaling across a notably broad range of cellular contexts.

Structurally, CD40L assembles as a homotrimer (4) and engages CD40 in a crevice formed between two adjacent subunits. The crystal structure of the complex (PDB: 3QD6, 3.5 Å resolution) shows that CD40 binds the trimeric interface asymmetrically, with charge complementarity as the primary determinant of binding specificity, so that several HIGM1-associated missense mutations map directly to this interface (5).

X-linked hyper-IgM syndrome (XHIGM), caused by mutations in CD40LG at chromosome Xq26.3, is characterized by defective T-B lymphocyte crosstalk and failure of class-switch recombination, resulting in low IgG, IgA and IgE with normal or elevated IgM (6). More than 50% of affected males develop symptoms by age one and more than 90% by age four (7). The clinical spectrum includes *Pneumocystis jirovecii* pneumonia, *Cryptosporidium* sp. infection, hepatobiliary complications, and hematological manifestations. Absent or nonfunctional CD40L disrupts costimulation of macrophages and dendritic cells, impairing T cell differentiation and host defense (6).

Inborn errors of immunity remain underdiagnosed in Latin America, partly because specialized diagnostic and functional validation assays are rarely accessible (7–9). HIGM1 is particularly difficult to identify in this setting, since its presentation overlaps considerably with common regional infections, and the functional consequences of CD40L variants found in Latin American patients are seldom characterized. Phenotypic and molecular studies of Mexican patients with HIGM1 have documented variants associated with absent or severely reduced CD40L surface expression and abrogated receptor-binding capacity (10). Computational structural approaches offer a complementary route for characterizing such variants when experimental validation is not feasible and may support improved variant classification and molecular diagnosis in resource-limited settings (11).

We selected three missense mutations previously reported in the literature or in clinical records. A123E was first identified by DiSanto et al. (12) in a patient with HIGM1 and shown experimentally to produce a CD40L molecule incapable of binding CD40 (12). S222F was reported by Lopez-Granados et al. (13) in a patient in whom flow cytometry demonstrated normal CD40L surface expression but complete absence of CD40 receptor-binding capacity (13); the same variant was subsequently found in Mexican patients with HIGM1 with comparable phenotypic findings (10). G257R was identified in a Mexican patient with severely reduced CD40L surface expression (10) and it had not been reported in the peer-reviewed literature prior to this work, so its structural and functional consequences were entirely unknown. All three variants are associated with the XHIGM phenotype, and their distinct physicochemical natures make them well suited to dissecting the structural mechanisms by which CD40L mutations cause immune dysfunction. We hypothesized that A123E, S222F and G257R impair CD40L function through distinct mechanisms affecting binding affinity, trimeric stability, or signaling competence, and that molecular dynamics simulations can generate mechanistic evidence to support the functional interpretation of CD40LG variants in patients.

Prior computational work combining AlphaFold2 structural modeling with atomistic MD simulations and MM-PBSA free energy calculations has proven effective for dissecting mutation effects in protein-protein complexes at atomic resolution (14). Here we apply this framework to the native and three mutant CD40L-CD40 complexes using PDB: 3QD6 as the primary starting structure; AlphaFold2 was used exclusively to reconstruct short missing loops (residues 106–111 in CD40 chains Q and R, and residues 180–187 in CD40L chains A-C) absent from the crystal structure (5, 14). Two independent 300 ns simulations were performed per system. Analyses included root mean square displacement (RMSD), root mean square fluctuations (RMSF), radius of gyration, contact maps, CD40L-CD40 contact areas, binding free energies (in frames sampled every 10 ns over the final 200 ns), and APBS electrostatic calculations (in frames sampled every 50 ns between 100 and 300 ns).

## 2 Materials and methods

### 2.1 Construction of the CD40L-CD40 complex

The starting structure was obtained from the Protein Data Bank (PDB: 3QD6, 3.5 Å resolution) (5). Visualization and initial structural analysis used VMD (15). Missing chain S and incomplete regions, namely residues 106–111 in chains Q and R of CD40 and residues 180–187 in chains A, B, and C of CD40L, were reconstructed by integrating AlphaFold2 predictions; the predicted structures for CD40L (UniProt: P29965) (16, 17) and CD40 (UniProt: A0A0S2Z3C7) (18, 19) were retrieved from the AlphaFold database and integrated into the complex, yielding a model spanning the extracellular domain of CD40L (residues 119 to 261) and the extracellular region of CD40 (residues 21 to 145).

### 2.2 Selection of the studied mutations

Three CD40L missense mutations were selected based on prior identification in the literature or clinical records and their physicochemically distinct nature.

A123E (p.Ala123Glu; NM_000074.3:c.368C>A; ClinVar VCV000011168) (20) was first reported by DiSanto et al. (12), who showed by flow cytometry that this change produces a CD40L molecule unable to bind CD40 (12). The variant is classified as Pathogenic in ClinVar and is absent from population frequency databases. It replaces a nonpolar alanine with a negatively charged glutamic acid at a conserved position.

S222F (p.Ser222Phe) was reported by Lopez-Granados et al. (13) in a patient showing normal CD40L surface expression but CD40-Fc binding completely absent, effectively a null phenotype (13). The same variant was identified in Mexican patients with HIGM1, where it was likewise associated with severely reduced CD40L surface expression and absent receptor-binding capacity (10). The substitution replaces a polar serine with a bulky aromatic phenylalanine at a position within the same beta strand as Gln220, in close proximity to the CD40-binding groove.

G257R (p.Gly257Arg) was identified in a Mexican patient with HIGM1 and severely reduced CD40L surface expression detected by flow cytometry (10). The variant has not been reported in peer-reviewed literature and had not been characterized at any structural or functional level before this study. It replaces glycine, the smallest and most conformationally flexible amino acid, with arginine, one of the largest and most positively charged residues, making it the most radical physicochemical substitution among the three variants.

### 2.3 Generation of mutant models

Point mutations were introduced using the psfgen module, generating one independent model per variant. Each mutant model was subsequently minimized following the same protocol as the wild-type. Stereochemical quality was assessed with the SwissModel server; only models with QMEAN below 2 and acceptable Ramachandran distributions were accepted for simulation.

### 2.4 Preparation of simulation boxes

Each system contained the CD40L-CD40 complex, TIP3P water molecules, and Na^+^ and Cl^-^ ions at 0.15 M NaCl to neutralize the system and approximate physiological ionic conditions. The systems were described using the CHARMM36 all-atom force field with CMAP corrections for all protein atoms (21, 22). The native CD40L-CD40 complex contained 12,048 protein atoms and 119,506 atoms in total after solvation. The A123E, S222F, and G257R variants contained 12,063, 12,075, and 12,099 protein atoms, respectively, corresponding to total system sizes of 119,518, 119,533, and 119,554 atoms. All systems were solvated in explicit TIP3P water and simulated under periodic boundary conditions in identical simulation boxes of 110 × 110 × 105 Å³.

### 2.5 Molecular dynamics simulations

All simulations were run with NAMD 2.14 (23) in the NPT ensemble, using Langevin dynamics for temperature control (300 K) and a Nose-Hoover Langevin piston barostat for pressure control (1 bar). Non-bonded interactions used a cutoff of 11.5 Å with a shifting function starting at 10.0 Å. Long-range electrostatics were computed with the Particle Mesh Ewald method (24). Hydrogen bonds were constrained with SHAKE (25). The integration timestep was 2 fs and coordinates were saved every 10 ps. Protonation states were assigned according to standard CHARMM36 titratable residue parameters at pH 7.0. Prior to production, each system underwent energy minimization followed by a six-stage equilibration protocol with positional restraints on protein heavy atoms, with force constants decreasing progressively from 15, 10, 5, 3, 2, to 1 kcal/mol·Å². Each system was then simulated for 300 ns in two independent replicates. RMSD analysis confirmed equilibration within the first 50 ns, and the final 200 ns were used for all analyses.

### 2.6 Trajectory analysis

Analyses over the final 200 ns were performed in the VMD environment and in-house Python scripts. Contact areas between the CD40L trimeric interface and the CD40L-CD40 intermolecular interface were calculated using a distance cutoff of 4.5 Å between protein heavy atoms. Contact maps were built by scoring residue-residue pairs by their temporal contact frequency across sampled frames, using the same 4.5 Å cutoff. Additional analyses included RMSD and RMSF of Cα atoms and the radius of gyration.

### 2.7 Binding free energy calculations

#### 2.7.1 MM-PBSA

Binding free energies for the full trimeric CD40L (treated as the ligand) bound to CD40 (receptor) were estimated with the GROMACS implementation of the MM-PBSA (26), as averages on conformations taken every 10 ns over the last 200 ns, for a total of 20 frames per system per replicate. The binding free energy was broken down into molecular mechanics contributions (electrostatic and van der Waals), polar solvation energy from the Poisson-Boltzmann equation, and non-polar solvation energy from the solvent accessible surface area (SASA) using a single linear term (inp = 1; γ = 0.005 kcal/mol/Å², offset = 0.000), with an internal dielectric constant of 1.0 and an external dielectric constant of 80.0 at an ionic strength of 0.15 M. Entropic contributions (−TΔS) were estimated using the quasiharmonic approximation implemented in gmx_MMPBSA and are reported in **Table 1**. The values were comparable across all systems, supporting that entropic differences do not substantially alter the energetic ranking; however, we acknowledge that this assumption may not hold for mutations producing large conformational rearrangements (27). Per-residue contribution was calculated to identify interface hotspots. Results are reported as mean ± SD/SEM across the 20 frames. All comparisons between systems are descriptive; the SD and SEM reported characterize within-system variability across frames and are not intended to support formal statistical inference between conditions.

**Table 1:** MM-PBSA binding free energies (kcal/mol) for each system in both replicates.

R	System	ΔVdW	ΔE_EL_	ΔE_PB_	ΔE_NP_	ΔG_GAS_	ΔG_SOLV_	ΔH_bind_	-TΔS	ΔG_bind_	SD	SEM
1	Native	-213.47	-4554.43	4701.40	-32.61	-4767.90	4668.79	-99.11	104.81	5.70	42.52	9.06
1	A123E	-217.06	-3725.54	3889.21	-33.15	-3942.59	3856.06	-86.53	105.20	18.67	50.20	10.70
1	S222F	-178.61	-4723.53	4884.59	-39.90	-4902.13	4844.70	-57.44	99.22	41.78	77.14	17.25
1	G257R	-208.23	-5466.52	5622.89	-36.81	-5674.74	5586.08	-88.66	106.95	18.28	98.08	20.91
2	Native	-225.75	-4668.25	4848.90	-37.39	-4894.00	4811.52	-82.48	104.79	22.31	68.77	14.66
2	A123E	-299.90	-3890.48	4078.33	-35.32	-4120.37	4043.01	-77.37	105.93	28.57	46.67	9.95
2	S222F	-228.77	-4735.51	4932.30	-37.00	-4964.28	4895.30	-68.98	105.45	36.46	63.94	13.63
2	G257R	-195.29	-5738.07	5879.43	-34.67	-5933.37	5844.76	-88.60	105.33	16.73	43.46	9.26

R, replicate; System, analyzed system; ΔVdW, van der Waals energy contribution; ΔE_EL_, electrostatic energy contribution; ΔE_PB_, polar solvation energy calculated using the Poisson–Boltzmann equation; ΔE_NP_, non-polar solvation energy contribution; ΔG_GAS_, total gas-phase energy; ΔG_SOLV_, total solvation energy; ΔH_bind_, enthalpic contribution to binding; −TΔS, entropic contribution; ΔG_bind_, total binding free energy; SD, standard deviation; SEM, standard error of the mean. Energy values are expressed in kcal/mol.

#### 2.7.2 APBS electrostatic analysis

**Figure 1:**
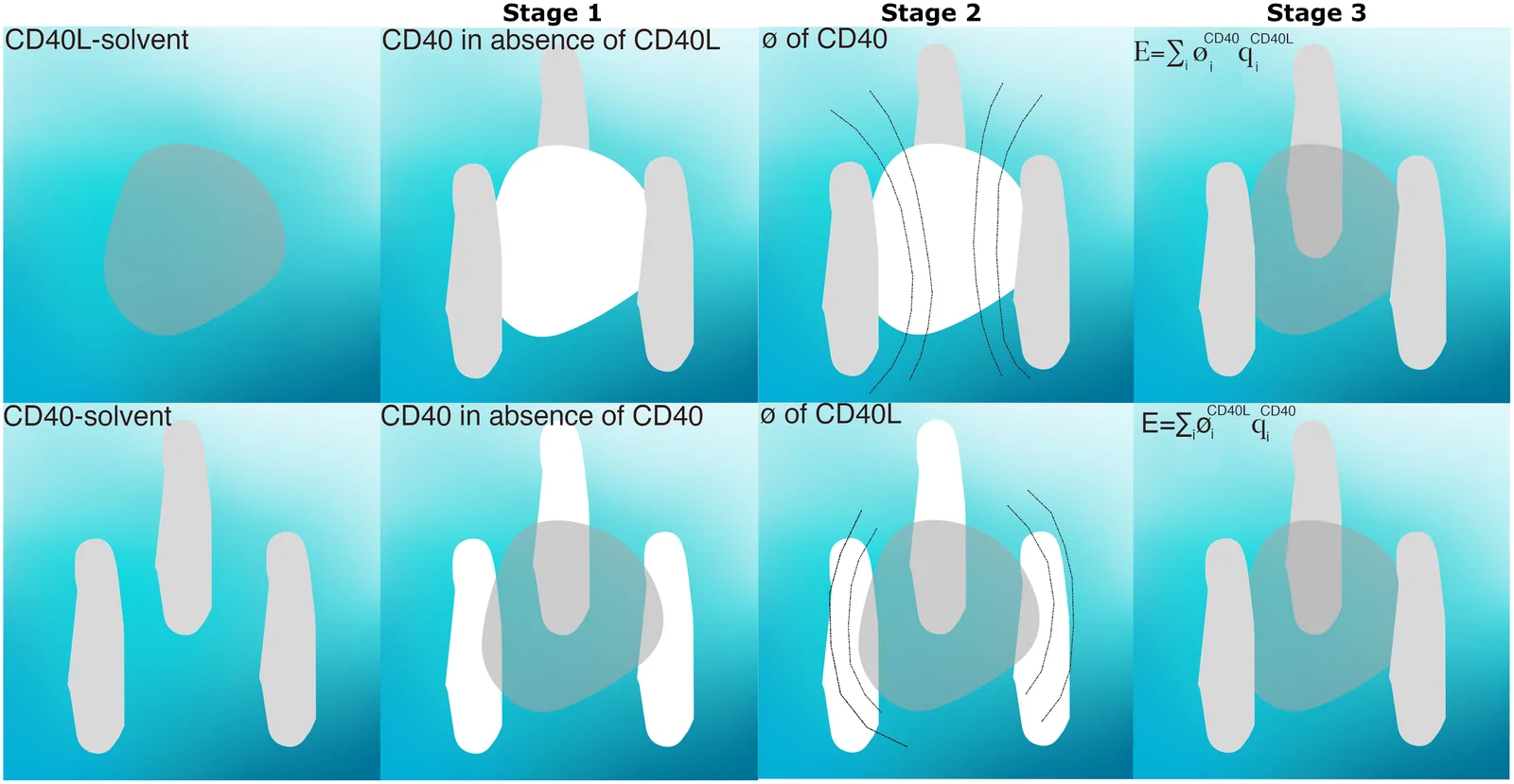
Calculation of the electrostatic binding interaction in the complex CD40L-CD40. Three-stage workflow for computing the CD40L-CD40 electrostatic interaction energy, shown from two perspectives: CD40L-centered (top) and CD40-centered (bottom). Stage 1 depicts the desolvation of each protein in the absence of its partner, with translucent gray volumes indicating the displaced solvent. Stage 2 shows the electrostatic potential (Φ) projected onto the binding interface, represented by dashed field lines. Stage 3 yields the interaction energy as E = Σ_i_ Φ_i_^CD40^ q_i_^CD40L^ (top) and E = Σ_i_ Φ_i_^CD40L^ q_i_^CD40^ (bottom), weighting each protein’s potential by the partial charges of its partner.

A specific analysis of the electrostatic interactions in the CD40L-CD40 complex was performed with the adaptive Poisson-Boltzmann solver (APBS) (28). Representative configurations were obtained from MD trajectories with explicit solvent at 300 K, 1 bar, and 0.15 M NaCl. The electrostatic analysis proceeded in stages (**Figure 1**) (29): first, the desolvation energy, computed as the difference of the energy of the complex in the solvent and the CD40L or CD40 (first partner) in the absence of the second partner: desolvation energy ΔG^CD40L/CD40^; second, calculation of the electric potential of one partner in absence of the second partner: Φi^CD40L/CD40^; third, the electrostatic interaction energy between CD40 and CD40L calculated from the electric potential of the first partner and the charges on the second partner: E=∑_i_Φ_i_^CD40L/CD40^q_i_^CD40/CD40L^. The first stage accounts for the energy cost of removing solvent-first partner interactions upon complex formation; the last stage captures the direct electrostatic coupling between both molecules in the complex CD40L-CD40.

## 3 Results

### 3.1 Structural stability and global conformational changes

**Figure 2:**
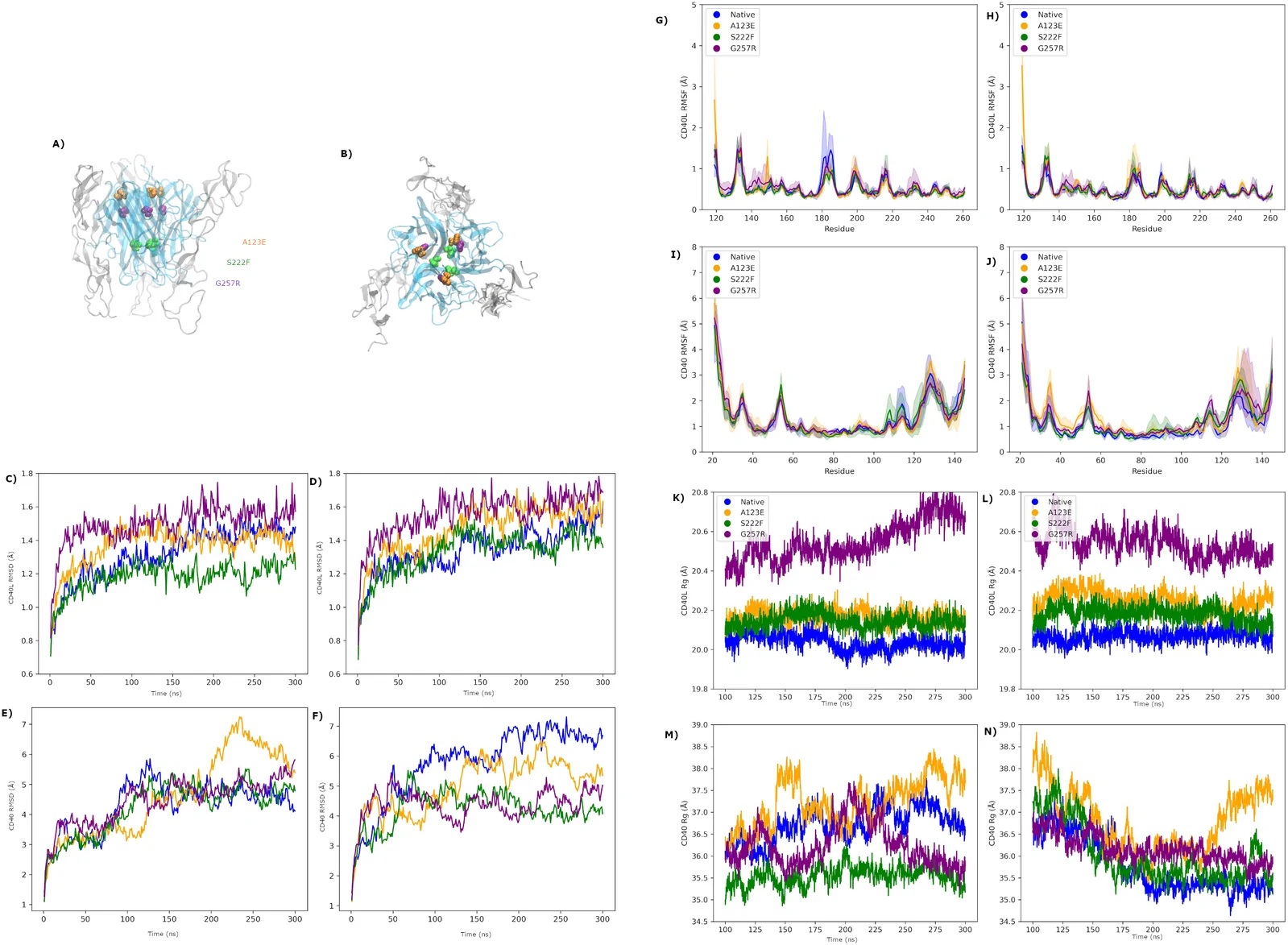
Structural dynamics of the CD40L-CD40 complex during molecular dynamics simulations. **(A)** Representative structure of the trimeric CD40L (light blue) in complex with CD40 (gray), with mutation sites highlighted as spheres (native system). **(B)** Alternative view of the complex showing the spatial arrangement of the three CD40L chains and their interaction with CD40. **(C, D)** Root mean square deviation (RMSD, Å) of CD40L as a function of simulation time (ns) for replicate 1 **(C)** and replicate 2 **(D)**, for the native system (blue), A123E (orange), S222F (green), and G257R (purple) mutants. **(E, F)** RMSD (Å) of CD40 as a function of simulation time for replicate 1 **(E)** and replicate 2 **(F)**. **(G, H)** Root mean square fluctuation (RMSF, Å) per residue of CD40L for replicate 1 **(G)** and replicate 2 **(H)**, illustrating per-residue flexibility across systems. **(I, J)** RMSF (Å) per residue of CD40 for replicate 1 **(I)** and replicate 2 **(J)**. **(K, L)** Radius of gyration (Å) of CD40L as a function of simulation time for replicate 1 **(K)** and replicate 2 **(L)**. **(M, N)** Radius of gyration (Å) of CD40 as a function of simulation time for replicate 1 **(M)** and replicate 2 **(N)**. Color code: native (blue), A123E (orange), S222F (green), G257R (purple).

The structure of the CD40L-CD40 complex used as the initial configuration for all simulations is shown in **Figures 2A, B**. The trimeric CD40L (light blue) is shown in complex with CD40 (gray), with the three mutation sites highlighted as spheres at their respective positions within the trimeric scaffold. The two views convey the overall architecture of the complex and the spatial distribution of the mutated residues relative to the CD40L homotrimer core and the CD40-binding interface.

RMSD analysis confirmed that all systems equilibrated within the first 50 ns of both 300 ns replicates and remained stable thereafter. The elevated RMSD of CD40 relative to CD40L reflects increased mobility at the terminal regions of the ectodomain, which in the full-length receptor would be constrained by the transmembrane domains; this is an expected consequence of the truncated model, and does not affect the interactions in the complex (**Figures 2C–F**).

RMSF analysis showed reproducible flexible regions of the CD40L and CD40 (**Figures 2G, H**); the main difference between replicate 1 and 2 of CD40L was in the native system relative to the three mutants in residues 180 to 190. Small differences in the flexibility of the mutants and the native suggest that the mutations reorganize local dynamics in this region. No clear RMSF differences were observed for CD40 in either replicate (**Figures 2I, J**).

Radius of gyration (Rg) for CD40 spread in the interval 35 to 38 Å without differential patterns (**Figures 2M, N**). For CD40L, however, G257R consistently displayed a markedly higher Rg than the native and the other two mutants in both independent replicates, indicating a global structural expansion of the trimeric ligand (**Figures 2K, L**). Neither A123E nor S222F produced this effect, pointing to a specific consequence of the G257R substitution on the organization of the trimeric scaffold.

### 3.2 Local contact environment of the mutated residues

**Figure 3:**
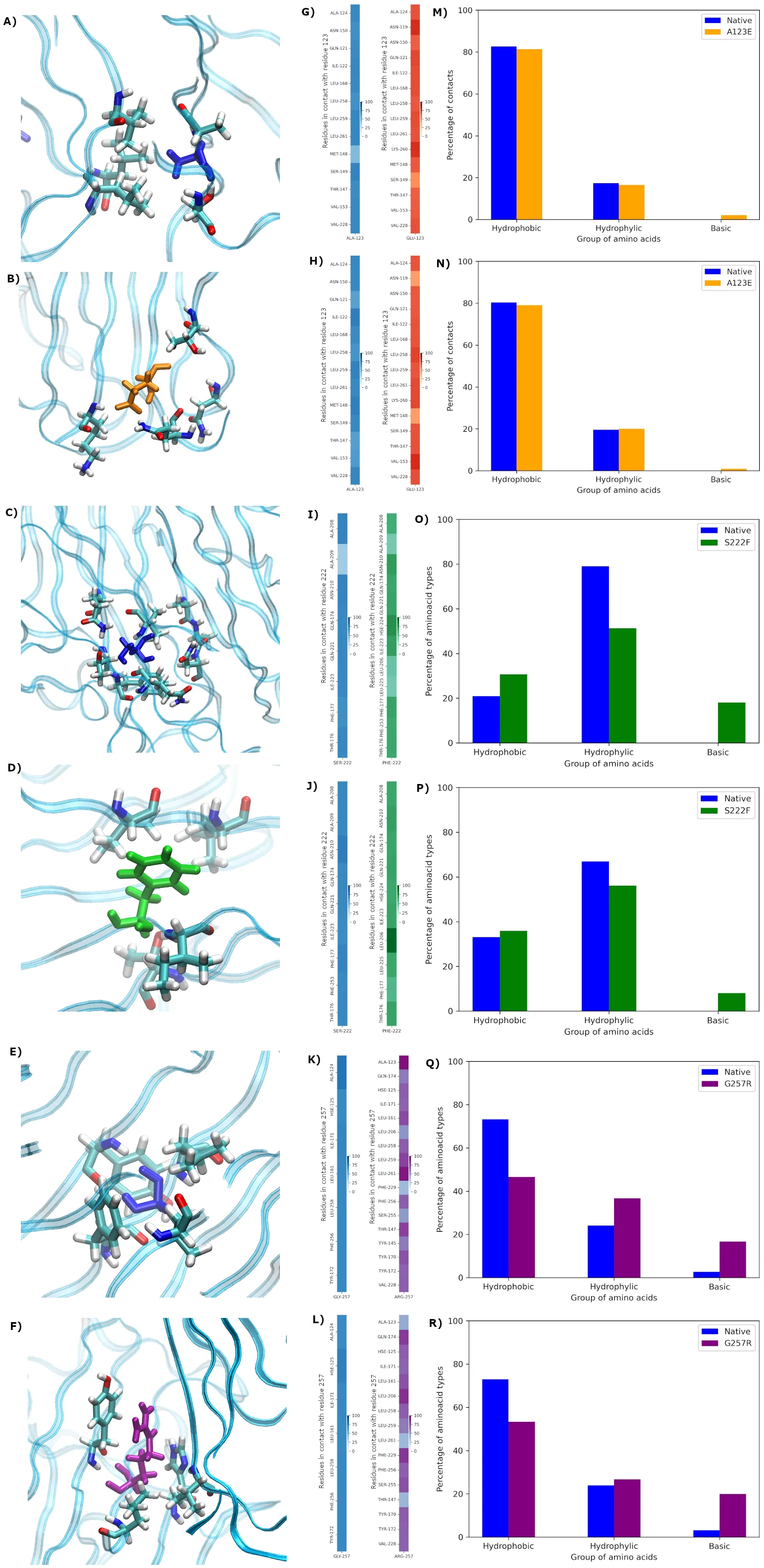
Interface residue contacts and physicochemical characterization of the CD40L-CD40 interaction surface. **(A, B)** Close-up view of the interaction interface at residue 123 in the native **(A)** and A123E mutant **(B)** systems, showing the local rearrangement of contacts induced by the mutation. **(C, D)** Interaction interface at residue 222 in the native **(C)** and S222F mutant **(D)** systems. **(E, F)** Interaction interface at residue 257 in the native **(E)** and G257R mutant **(F)** systems. **(G-L)** Percentage of intermolecular contacts established throughout the simulation trajectory at the CD40L-CD40 interface for the native system at residue 123 **(G)**, A123E mutant **(H)**, native system at residue 222 **(I)**, S222F mutant **(J)**, native system at residue 257 **(K)**, and G257R mutant **(L)**; color intensity reflects contact frequency. **(M, N)** Percentage of contacts classified by amino acid group (hydrophobic, hydrophilic, and basic) at the interface of the native and A123E systems for replicate 1 **(M)** and replicate 2 **(N)**. **(O, P)** Equivalent contact classification for the native and S222F systems, replicate 1 **(O)** and replicate 2 **(P)**. **(Q, R)** Contact classification for the native and G257R systems, replicate 1 **(Q)** and replicate 2 **(R)**. Color code: native (blue), A123E (orange), S222F (green), G257R (purple).

At position 123, A123E increased the number of residues in contact with the mutated site relative to native alanine in both replicates (**Figures 3A, B**). The native structure showed a compact local environment around alanine 123. In the A123E system, the negatively charged glutamic acid side chain appears to be surrounded by more residues, including additional basic contacts (**Figures 3G, H, M, N**).

At position 222, S222F increased the number of contacting residues, with a shift toward hydrophobic contacts and the appearance of basic residues nearby (**Figures 3C, D**). The native serine at this position showed a predominantly polar local environment. In the S222F system, the phenylalanine side chain occupies a larger local volume and is associated with a higher proportion of hydrophobic contacts in both replicates (**Figures 3I, J, O, P**).

At position 257, G257R showed the largest local contact rearrangement among the three variants (**Figures 3E, F**). Compared with native glycine, the arginine residue occupies a larger local volume and is associated with an expanded contact shell, fewer hydrophobic contacts, and more hydrophilic and basic contacts in both replicates (**Figures 3K, L, Q, R**).

### 3.3 Contact map analysis of the CD40L-CD40 binding interface

**Figure 4:**
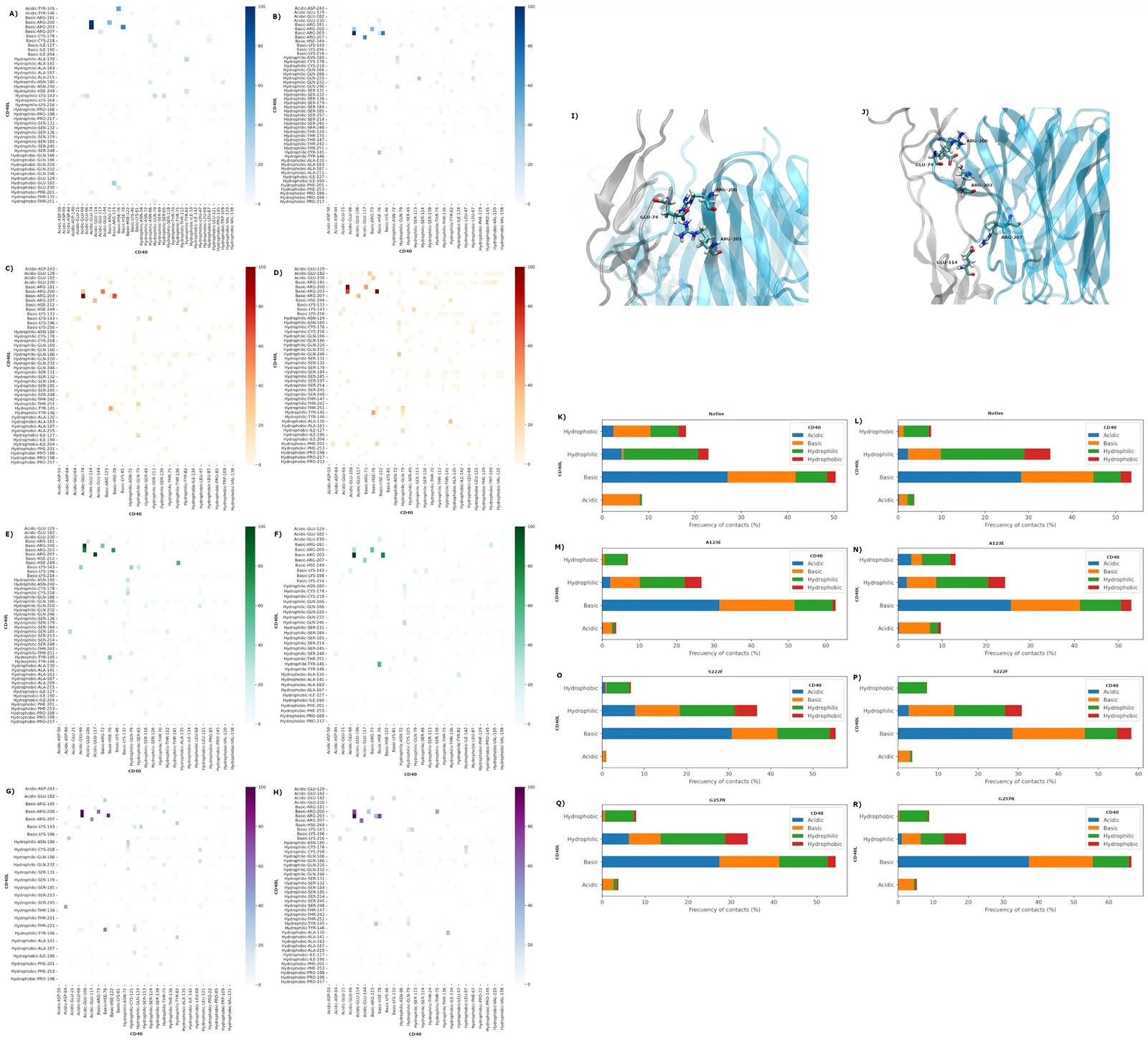
Intermolecular contact maps and interface interaction profiles of the CD40L-CD40 complex. **(A, B)** Residue-residue contact maps between CD40L and CD40 for the native system, replicate 1 **(A)** and replicate 2 **(B)**; color intensity indicates contact frequency. **(C, D)** Contact maps for the A123E mutant, replicate 1 **(C)** and replicate 2 **(D)**. **(E, F)** Contact maps for the S222F mutant, replicate 1 **(E)** and replicate 2 **(F)**. **(G, H)** Contact maps for the G257R mutant, replicate 1 **(G)** and replicate 2 **(H)**. **(I)** Representative structure of the CD40L-CD40 interface in the native system highlighting the key electrostatic interactions between ARG-200, ARG-203 (CD40L) and GLU-74 (CD40). **(J)** Native system interface showing the same ARG-200/ARG-203-GLU-74 interactions alongside additional strong contacts involving ARG-207 and GLU-114. **(K, L)** Frequency of contacts (%) at the CD40L-CD40 interface classified by amino acid type (hydrophobic, hydrophilic, basic, and acidic) for the native system, replicate 1 **(K)** and replicate 2 **(L)**. **(M, N)** Contact frequency profiles for the A123E mutant, replicate 1 **(M)** and replicate 2 **(N)**. **(O, P)** Contact frequency profiles for the S222F mutant, replicate 1 **(O)** and replicate 2 **(P)**. **(Q, R)** Contact frequency profiles for the G257R mutant, replicate 1 **(Q)** and replicate 2 **(R)**.

In both replicates, the most frequent contact at the binding interface involved a basic arginine from CD40L and an acidic glutamic acid from CD40: ARG-200 and GLU-74 in replicate 1, and ARG-203 and GLU-74 in replicate 2 (**Figures 4I, J**). The structural representations show ARG-200 and ARG-203 within an arginine-rich region in close proximity to GLU-74 during the simulations. Additional contacts, including ARG-207 and GLU-114, were observed at lower frequencies in replicate 2.

Secondary contact frequencies differed across systems. The native system showed one predominant and one secondary contact pair (**Figures 4A, B**). A123E showed increased frequencies of secondary arginine-mediated contacts and additional residue pairs (**Figures 4C, D**). S222F showed a more even distribution of contact frequencies among ARG-200, ARG-203, and ARG-207 (**Figures 4E, F**). G257R showed lower frequencies of secondary contacts while retaining the predominant ARG/GLU contact, resulting in fewer high-frequency residue pairs in both replicates (**Figures 4G, H**).

Basic residues from CD40L accounted for approximately 50-65% of the contact frequency across all systems and replicates and interacted predominantly with acidic residues from CD40 (**Figures 4K, L**). A123E and G257R showed the most similar interfacial residue-class distributions between replicates (**Figures 4M, N, Q, R**). S222F showed the largest difference between replicates, with a higher proportion of hydrophobic contacts in replicate 2 than in replicate 1 (**Figures 4O, P**). In both replicates, the proportion of basic-acidic contacts was lower for S222F than for the native system.

### 3.4 Contact area analysis

**Figure 5:**
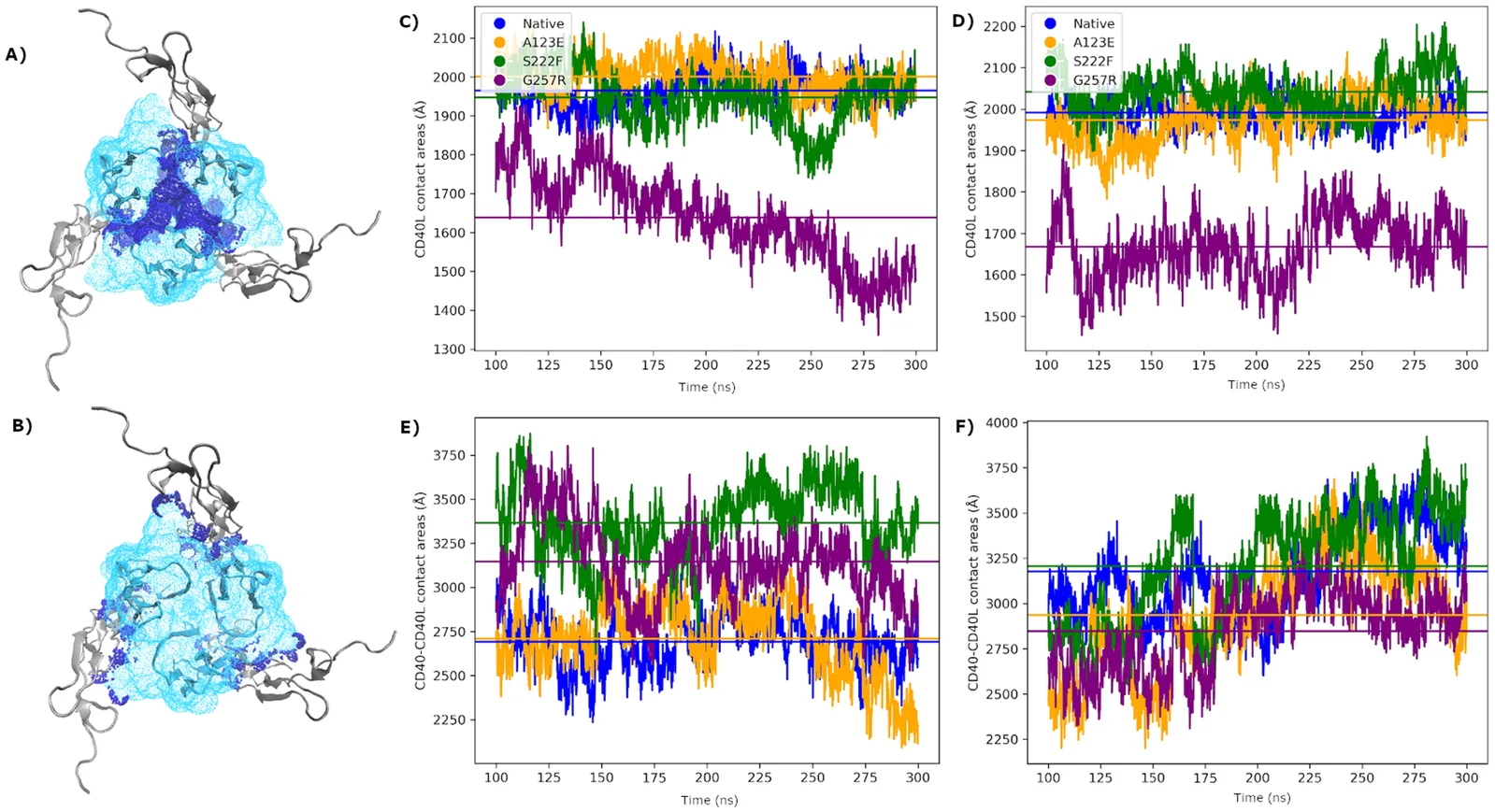
Solvent-accessible surface area and interface contact areas of the CD40L-CD40 complex. **(A)** Surface representation of the CD40L homotrimer; the dark blue region highlights the inter-chain contact area between CD40L subunits. **(B)** Surface representation of the CD40L-CD40 complex; the dark blue region indicates the interface contact area between CD40L and CD40. **(C, D)** Time evolution of the CD40L inter-chain contact area (Å²) for replicate 1 **(C)** and replicate 2 **(D)**, comparing the native system (blue), A123E (orange), S222F (green), and G257R (purple) mutants; horizontal lines indicate the mean value for each system. **(E, F)** Time evolution of the CD40L-CD40 interface contact area (Å²) for replicate 1 **(E)** and replicate 2 **(F)**; horizontal lines indicate the mean value for each system. Color code: native (blue), A123E (orange), S222F (green), G257R (purple).

The surface representations in **Figures 5A, B** illustrate the geometric basis for the contact area calculations. The dark blue region in **Figure 5A** delineates the inter-chain contact surface between CD40L subunits within the homotrimer, while **Figure 5B** shows the intermolecular contact surface between CD40L and CD40.

At the CD40L trimeric interface, native, A123E and S222F all maintained comparable contact areas (approximately 1947-2042 Å² across replicates) (**Figures 5C, D**). G257R showed a markedly reduced trimeric contact area in both replicates (1554 ± 136 Å² in R1; 1667 ± 74 Å² in R2), corresponding to a reduction of 20-22% relative to the native (1972-1994 Å²). The higher standard deviation for G257R in replicate 1 further reflects structural variability at this interface.

At the CD40L-CD40 intermolecular interface, A123E maintained contact areas comparable to the native in both replicates (**Figures 5E, F**). S222F showed a consistent increase across both replicates (3341 ± 195 Å² in R1; 3195 ± 269 Å² in R2) relative to native (2658 ± 153 and 3218 ± 232 Å²), indicating that the phenylalanine expands the interface geometrically without improving energetic quality. G257R showed intermediate values (3012 ± 250 and 2919 ± 223 Å²) without a clear directional change. The key distinction is that G257R selectively compromises the trimeric interface while leaving the receptor-binding surface area largely unaltered, whereas S222F expands the CD40L-CD40 contact area without improving binding affinity.

### 3.5 Binding free energy calculations

#### 3.5.1 MM-PBSA

Binding free energies calculated from 20 frames per system per replicate are summarized in **Table 1**. The native complex showed ΔG values of 5.70 and 22.31 kcal/mol in replicates 1 and 2, respectively. The positive absolute ΔG values may result from the quasiharmonic approximation overestimating the configurational entropy for large complexes that sample multiple energy wells (27). Accordingly, these positive values are not interpreted as absolute measures of binding favorability or as evidence that complex formation is thermodynamically unfavorable; only relative differences among systems calculated using the same protocol are considered.

A123E showed ΔG values of 18.67 and 28.57 kcal/mol in replicates 1 and 2, respectively. S222F showed the highest ΔG values among the four systems, at 41.78 and 36.46 kcal/mol. G257R showed ΔG values of 18.28 and 16.73 kcal/mol, which were closer to the corresponding native values than those obtained for S222F. The individual energetic components showed broadly similar patterns across the two replicates, with G257R displaying particularly similar ΔEEL, ΔHbind, −TΔS, and ΔG values. These comparisons are descriptive; the reported SD and SEM characterize within-system variability across frames and are not intended to support formal statistical comparisons between systems.

#### 3.5.2 Electrostatics in the complex: APBS calculations

The APBS calculations were performed mainly to evaluate the electrostatic interactions between CD40L-CD40, without the van der Waals repulsion/dispersion contributions. It is important to note that APBS computes only electrostatic solvation free energies (desolvation penalty plus Coulombic interaction), whereas MM-PBSA additionally includes van der Waals, polar solvation, and non-polar solvation contributions. Divergence between the two methods therefore reflects the relative magnitude of non-electrostatic contributions to binding. Results from five frames per system per replicate are summarized in **Table 2**. The native complex showed a ΔG electrostatic of -126.02 and -83.48 kcal/mol, respectively, in replicate 1 and 2. The stronger electrostatic interactions observed in the mutants correlate with an increase in the number of contacts between CD40L and CD40 (**Figures 4B, C**). In the native complex, the average total number of contacts was 182 across two replicates, corresponding to a ΔG electrostatic of −126.02 and −83.48 kcal/mol. The G257R mutant showed the highest electrostatic affinity of all systems, with a ΔG electrostatic of −140.47 and −146.19 kcal/mol in replicates 1 and 2, respectively, which is consistent with the largest number of contacts observed, averaging 250 across replicates. This enhancement is likely attributable to the positively charged arginine residue, which increases electrostatic complementarity with CD40. In contrast, the A123E mutant displayed a moderate improvement in electrostatic interactions (-85.96 and −100.76 kcal/mol, respectively, in replicate 1 and 2) accompanied by a reduction in the number of contacts to an average of 202, reflecting a weaker but still favorable electrostatic contribution relative to the native complex. Similarly, S222F showed intermediate electrostatic affinity (−132.01 and −117.38 kcal/mol in replicates 1 and 2, respectively) with an average contact number of 179, comparable to the native complex. These results suggest a consistent trend in which more favorable electrostatic solvation free energies are associated with a greater number of interfacial contacts between CD40L and CD40, highlighting the role of electrostatic complementarity in stabilizing the protein-protein interaction.

**Table 2:** APBS electrostatic solvation free energies (kcal/mol) for each system in both replicates.

R	System	CD40L	CD40	Complex (CD40L without charge)	Complex (CD40 without charge)	CD40L desolvated	CD40 desolvated	Energy of CD40L in the CD40 field	Energy of CD40 in the CD40L field	ΔG electrostatic	SD ΔG electrostatic
1	Native	9.05E+04	8.41E+04	8.42E+04	9.07E+04	157.53	152.81	-218.07	-218.29	-126.02	29.83
1	A123E	9.06E+04	8.35E+04	8.37E+04	9.08E+04	157.95	148.21	-195.97	-196.15	-85.96	29.20
1	S222F	9.30E+04	8.66E+04	8.68E+04	9.32E+04	186.68	168.43	-243.57	-243.55	-132.01	42.82
1	G257R	9.19E+04	8.43E+04	8.45E+04	9.20E+04	180.77	158.92	-239.88	-240.28	-140.47	32.62
2	Native	8.70E+04	8.08E+04	8.10E+04	8.71E+04	172.37	171.39	-213.58	-213.65	-83.48	32.64
2	A123E	8.88E+04	8.19E+04	8.21E+04	8.90E+04	190.28	167.64	-229.50	-229.18	-100.76	35.36
2	S222F	8.80E+04	8.20E+04	8.21E+04	8.82E+04	189.13	173.56	-239.99	-240.07	-117.38	23.78
2	G257R	8.92E+04	8.18E+04	8.20E+04	8.93E+04	161.99	145.73	-226.87	-227.04	-146.19	35.76

R, replicate; System, analyzed system; CD40L, electrostatic free energy of CD40L; CD40, electrostatic free energy of CD40; Complex (CD40L without charge), electrostatic free energy of the complex calculated with CD40L charges set to zero; Complex (CD40 without charge), electrostatic free energy of the complex calculated with CD40 charges set to zero; CD40L desolvated, desolvation energy of CD40L upon complex formation; CD40 desolvated, desolvation energy of CD40 upon complex formation; Energy of CD40L in the CD40 field, interaction energy of CD40L in the electrostatic field generated by CD40; Energy of CD40 in the CD40L field, interaction energy of CD40 in the electrostatic field generated by CD40L; ΔG electrostatic, total electrostatic free energy of binding; SD ΔG electrostatic, standard deviation of the electrostatic binding free energy. Energy values are expressed in kcal/mol.

Reading MM-PBSA and APBS together reveals distinct profiles for each mutant. For A123E, both methods indicate a coherent reduction in binding affinity, consistent with moderate and straightforward loss of binding competency. For S222F, the two methods diverge: it is the weakest MM-PBSA binder yet shows improved APBS, indicating that favorable electrostatic reorganization is more than offset by van der Waals and solvation penalties from the bulky side chain. For G257R, near-native MM-PBSA combined with enhanced APBS points to a mutation that does not weaken binding energetically yet produces the most extensive structural reorganization of the trimer.

## 4 Discussion

In this study we describe global and local impacts of missense mutations of the CD40L in complex with CD40 using molecular dynamics simulations and free energy calculations. All mechanistic conclusions presented herein are computational predictions and require experimental validation (including binding assays, surface expression studies, and signaling readouts) to be confirmed. In addition, the energetics in the interaction was evaluated in terms of time frames from the two independent 300 ns long simulation trajectories per system, using MM-PBSA (26, 30) and APBS (28). The central finding is that each mutation is predicted to perturb the CD40L-CD40 complex through a distinct structural reorganization, providing a candidate mechanistic framework for the functional and clinical heterogeneity of HIGM1. CD40L functions as a homotrimer (31, 32) that engages CD40 on B cells and antigen-presenting cells, initiating NF-κB, MAPK and PI3K signaling cascades and driving class-switch recombination, somatic hypermutation and germinal center formation (33, 34). The downstream consequence of impaired CD40 signaling is failure to induce activation-induced cytidine deaminase (AID) via NF-κB, an enzyme strictly required for both CSR and somatic hypermutation in germinal center B cells. Importantly, the quantity and quality of CD40 signaling shape B cell fate decisions through NF-κB-dependent transcriptional programs, so that suboptimal engagement is insufficient for germinal center entry even when the interaction is not fully abrogated (33, 35). Functional CD40L therefore requires two structural prerequisites: a stable and properly organized homotrimer, and a productive binding interface with CD40. The three mutations studied here are predicted to compromise one or both of these prerequisites through different mechanisms, which we discuss in turn below.

### 4.1 A123E: Partial loss of binding affinity with limited structural disruption

A123E produced the most limited perturbation among the three variants. The trimeric contact area was unaffected, the radius of gyration was comparable to that of the native (**Figures 2K, L**), and both MM-PBSA and APBS indicated only a moderately lower binding affinity. The introduction of a negative charge at position 123 may enhance H-bonds or the electrostatic complementarity with ASN119, ASN150, GLN121, LYS260 of CD40. An increase of electrostatic attraction may induce closer contacts in the complex, but not necessarily increase the free energy binding, which also includes van der Waals repulsion/dispersion energies. The lower binding energy is consistent with partial or attenuated loss of CD40L function rather than outright abolition. Prior bioinformatic analyses have shown that CD40L mutations associated with partial or residual binding capacity tend to be linked to milder immunological phenotypes (36, 37), which fits the energetic profile of A123E. Even a partial impairment of the CD40L-CD40 interaction would be expected to compromise the efficiency of CSR, since the threshold of CD40 signaling required for B cell activation and germinal center reactions is sensitive to both the quality and the duration of the contact.

### 4.2 S222F: Steric disruption expands the interface while reducing binding affinity

S222F showed the weakest binding of the three mutants in both replicates, with the highest (least favorable) ΔGbind among all systems (41.78 and 36.46 kcal/mol vs. 5.70 and 22.31 for the native), in agreement with the functionally null phenotype documented experimentally by Lopez-Granados et al. (2003) (13) and reproduced in Mexican patients (10). The phenylalanine expands the CD40L-CD40 contact area (**Figures 5E, F**) while simultaneously reducing binding quality, consistent with a less complementary interface where the aromatic ring forces additional surface exposure without generating productive contacts (**Figures 3C, D, I, J**). The APBS calculation for S222F shows favorable electrostatic reorganization that nonetheless does not translate into stronger binding, a pattern previously described for protein-protein complexes in which electrostatic gains are offset by unfavorable van der Waals and solvation contributions (28). It is also worth noting that enhanced electrostatic complementarity at the interface, as seen with S222F, would slow CD40 dissociation and thereby restrict the lateral receptor mobility that is needed for productive CD40 clustering and signaling; work on CD40-targeting antibodies has shown that reduced affinity can paradoxically enhance agonistic activity precisely by facilitating receptor clustering (38, 39).

### 4.3 G257R: Trimeric destabilization may uncouple binding from signaling competence

G257R maintained near-native MM-PBSA binding free energies in both replicates and exhibited the most favorable APBS electrostatics of all systems, yet produced the most extensive structural reorganization of the four. This apparent paradox may be explained by three convergent mechanisms that we propose on the basis of the structural data. First, G257R reduced the CD40L trimeric interface area by 20-22% in both replicates (**Figures 5C, D**) alongside a consistently increased radius of gyration (**Figures 2K, L**), indicating structural loosening of the homotrimer. Since CD40L must assemble as a trimer to simultaneously engage three CD40 molecules and induce receptor clustering for TRAF recruitment and NF-κB activation (1, 40, 41), a disorganized trimer would fail to drive the precise receptor clustering required for downstream signaling even when individual pairwise contacts with CD40 remain energetically favorable. This point matters because multimeric forms of CD40L homotrimers are more active than homotrimers alone, underscoring how critically signal output depends on trimeric integrity. Second, the enhanced electrostatic complementarity of G257R would slow CD40 dissociation, restricting lateral receptor mobility and impairing the dynamic CD40 clustering required for efficient signal transduction (38, 39). Third, CD40 has been identified as a mechanosensor that forms catch bonds with CD40L, interactions paradoxically stabilized by mechanical force during the immunological synapse, and XHIGM-associated CD40L mutations have been shown to impair catch bond formation and force-enhanced CD40 signaling (42). The structural expansion and trimeric disorganization produced by G257R (**Figures 2K, L**, **4G, H**, **5C, D**) would compromise force transmission across the receptor-ligand interface, impairing the mechanosignaling required for sustained CD40 engagement. The severely reduced CD40L surface expression observed in the Mexican patient carrying G257R (10) is consistent with a mutation that destabilizes the trimeric scaffold and potentially affects surface trafficking or homotrimer stability in addition to signaling competence.

### 4.4 Clinical implications and relevance for Latin America

The three mutations studied here illustrate structurally distinct, computationally predicted categories of CD40LG dysfunction: partial binding affinity reduction (A123E), severe interface disruption with near-abolition of binding (S222F), and trimeric destabilization that we propose may uncouple binding from signaling (G257R). **Table 3** summarizes the mechanistic profile of each variant. This predicted mechanistic heterogeneity may provide a structural rationale for the variable severity, age of onset and residual immunoglobulin production observed across HIGM1 patients with different CD40LG mutations (36, 37, 43). Inborn errors of immunity remain significantly underdiagnosed in Latin America, where registries such as LASID (44) and LAGID (8, 9) have documented disparities in access to diagnosis and functional validation assays, highlighting the need for computational approaches that can provide mechanistic insight without requiring specialized experimental infrastructure.

**Table 3:** Mechanistic summary of the three CD40LG variants studied.

Mutation	Structural mechanism	Energetic signature	Predicted functional consequence
*A123E*	Moderate local electrostatic perturbation; interface architecture largely preserved	Coherent reduction in both MM-PBSA and APBS relative to native	Partial loss of binding affinity; compatible with attenuated or variable phenotype
*S222F*	Steric disruption by bulky aromatic side chain; geometrically expanded but energetically inferior interface	Largest MM-PBSA reduction; paradoxical improvement in APBS electrostatics	Strongest reduction in binding affinity; impaired CD40 signaling intensity and duration
*G257R*	Global trimeric expansion; 20-22% reduction in trimeric contact area; sparser receptor-binding interface	MM-PBSA near-native; most favorable APBS electrostatics of all systems	Uncoupling of binding and signaling competence via trimeric destabilization, restricted CD40 receptor mobility, and impaired mechanotransduction

From a clinical perspective, G257R illustrates what computational structural approaches can contribute to the characterization of CD40LG variants found in populations where experimental validation is limited. The variant was identified in a Mexican patient, is absent from the peer-reviewed literature, and had no prior structural characterization of any kind. The results presented here are therefore the first mechanistic description of this variant and a basis for hypothesis generation and variant prioritization; they support, but do not replace, the experimental functional validation required for definitive variant classification. More broadly, this framework may support the interpretation of variants of uncertain significance identified in Latin American patients, where access to specialized functional assays is often restricted and molecular diagnosis of HIGM1 and other inborn errors of immunity remains a clinical challenge that is far from resolved.

### 4.5 Limitations of the study

Several limitations of this study should be acknowledged. First, loop reconstruction used AlphaFold2, which provides a single static conformation and may not represent all conformational states accessible upon binding; however, the global architecture was derived from the experimental crystal structure (PDB: 3QD6). Second, 300 ns per replicate may not fully explore the energy landscape of this large complex; enhanced sampling methods such as replica exchange MD or metadynamics would provide broader coverage. Third, the transmembrane and cytoplasmic domains of CD40L and CD40 were excluded from the model, and the truncated ectodomain may not fully recapitulate membrane-proximal dynamics. Fourth, statistical comparison between systems is descriptive; the number of frames per replicate does not provide sufficient power for formal hypothesis testing, and independent experimental measurements are required. Fifth, although the calculated entropic contributions (−TΔS) were comparable across systems, the overestimated contribution of the configurational entropy hints the rough conformational energy surface of the CD40L-CD40 complex, and the impact of point mutations that dramatically alter conformational flexibility. Sixth, all mechanistic interpretations are computational predictions that require experimental validation including functional binding assays, surface-expression studies, receptor clustering experiments, and signaling readouts before they can be considered established. So far, we explored the molecular relationship between CD40L-CD40 complex on phenotypical observations according to clinical reports of HIGM, in terms of the structure and energetics impacts.

## 5 Conclusions

We characterized three CD40LG missense mutations associated with HIGM1 using molecular dynamics simulations, contact map and interface area analysis, and MM-PBSA and APBS binding free energy calculations across two independent replicates. Each mutation is predicted to perturb the CD40L-CD40 complex through a distinct mechanism. A123E produces a moderate, energetically coherent reduction in binding affinity compatible with partial loss of function. S222F causes the largest reduction in binding free energy through steric disruption of the interface by the aromatic side chain, consistent with the experimentally documented abolition of CD40 binding capacity. G257R maintains near-native binding free energies but reduces the trimeric contact area by 20-22% and expands the radius of gyration, a structural signature consistent with loosening of the homotrimer that we propose could uncouple binding from signaling competence; the associated effects on CD40 receptor mobility and mechanotransduction are hypotheses derived from the structural data that remain to be tested experimentally.

These findings are consistent with a model in which HIGM1-associated CD40LG mutations need not uniformly abolish receptor binding but may instead disrupt higher-order structural and dynamic properties of the complex that are required for productive signal transduction. That predicted mechanistic diversity may offer a structural explanation for the clinical heterogeneity of HIGM1, and it has a practical implication: the same downstream immunodeficiency phenotype may arise through energetically and geometrically distinct routes, which may eventually matter for therapeutic targeting. The characterization of G257R, a novel variant identified in a Mexican patient, illustrates the value of this computational framework for the functional interpretation of CD40LG variants in Latin American and other underrepresented populations where experimental validation may be limited, and its potential contribution to improved molecular diagnosis of inborn errors of immunity.

## Data Availability

The original contributions presented in the studyare publicly available. This data can be found here: https://doi.org/10.6084/m9.figshare.33190983, https://doi.org/10.6084/m9.figshare.33191175, https://doi.org/10.6084/m9.figshare.33191178, https://doi.org/10.6084/m9.figshare.33191184.
